# Not seeing or feeling is still believing: conscious and non-conscious pain modulation after direct and observational learning

**DOI:** 10.1038/srep16809

**Published:** 2015-11-18

**Authors:** Natalia Egorova, Joel Park, Scott P. Orr, Irving Kirsch, Randy L. Gollub, Jian Kong

**Affiliations:** 1 Department of Psychiatry, Massachusetts General Hospital/Harvard Medical School, Charlestown, MA, USA; 2 Program in Placebo Studies and Therapeutic Encounter, Beth Israel Deaconess Medical Center/Harvard Medical School, Boston, MA, USA

## Abstract

Our experience with the world is shaped not only directly through personal exposure but also indirectly through observing others and learning from their experiences. Using a conditioning paradigm, we investigated how directly and observationally learned information can affect pain perception, both consciously and non-consciously. Differences between direct and observed cues were manifest in higher pain ratings and larger skin conductance responses to directly experienced cues. However, the pain modulation effects produced by conditioning were of comparable magnitude for direct and observational learning. These results suggest that social observation can induce positive and negative pain modulation. Importantly, the fact that cues learned by observation and activated non-consciously still produced a robust conditioning effect that withstood extinction highlights the role of indirect exposure in placebo and nocebo effects.

Human ability to observe and imitate conspecifics likely evolved to make pain not only a personal experience but also a socially relevant behavior that solicits help and enables learning in others^1^,^2^,^3^. Observing others in pain produces an immediate effect on the observer’s pain perception, mostly through affective and sensory mechanisms^4^,^5^,^6^,^7^,^8^,^9^,^10^,^11^. In addition to modulating pain perception at the time of observation, seeing others’ pain behavior can induce higher-order learning and can change pain perception beyond the observation period itself. A number of studies have shown that vicarious (observational) conditioning in which subjects observe a model’s distress response, rather than receiving a distressing stimulus themselves^12^,^13^,^14^,^15^, produces a conditioned skin conductance response similar to that produced by classical conditioning. A conditioning-induced decrease/increase in perceived stimulus intensity is considered to be a placebo/nocebo-like effect, similar to the effect of the expectation of pain relief or aggravation from the context surrounding medical treatment^16^,^17^,^18^,^19^,^20^,^21^. Several studies investigating pain modulation by social observation have demonstrated that social observation can elicit placebo analgesia^22^,^23^ or nocebo hyperalgesia^24^,^25^ in response to a given painful stimulus. In these studies, the effects of pain observation were elicited using consciously perceived cues. Yet, cues learned through observation might be accessed automatically and exert influence without conscious awareness.

Recent studies using direct conditioning revealed that once conditioned, the cues can produce analgesia or hyperalgesia even when presented subliminally^26^,^27^. The influence on pain perception of non-consciously perceived cues associated with observationally learned responses, however, remains unknown. Exploring and comparing pain perception and modulation achieved through direct and observational conscious and non-conscious exposure to visual cues, as well as assessing the robustness of the modulation effect, are important for evaluating the potential use of social observation paradigms for pain modulation.

The present study (Fig. 1A) examined the ability of subliminally and supraliminally presented conditioned pain cues, established using both direct and observational conditioning procedures, to influence pain perception and placebo/nocebo effects. During direct conditioning subjects saw 2 abstract cues that predicted either a low or high heat stimulus to their forearm. During observational conditioning, the same subjects watched a video showing a person experiencing direct conditioning with 2 new cues that predicted either a low or high stimulus (Fig. 1B). During the test session (Fig. 1C), a moderate heat pain stimulus was paired with the directly conditioned low (1) and high (2) cues and the observationally conditioned low (3) and high (4) cues, as well as with a new neutral (5) cue. All cues were presented for either 200 ms (supraliminal) or 33 ms followed by a mask appearing for 167 ms (subliminal). Subjects’ pain ratings as well as skin conductance responses to each cue/pain stimulus were assessed. Several extinction trials and another test session were administered to assess robustness of the placebo/nocebo effects (Fig. 1A).

## Materials and Methods

### Subjects

Twenty-one healthy participants with no psychiatric or neurological disorders and not taking any psychotropic medication were enrolled. One subject did not complete all experimental sessions and was excluded from the study (before the test sessions). The final sample consisted of 20 participants (12 females) with an average age of 23 ± 2 years (Mean ± SD). The Institutional Review Board at Massachusetts General Hospital approved all study procedures. The study was conducted in accordance with the approved guidelines. All enrolled subjects provided written informed consent before beginning any study procedures.

### Stimuli and equipment

#### Visual Stimuli

A set of fractal images was used as visual cues. Each cue was associated with one of the five experimental conditions: a high direct cue and a low direct cue were first presented during the direct conditioning sessions and associated with a high or low level of heat pain respectively; a high observational cue and a low observational cue were presented in the observational conditioning sessions and were associated with high observed pain and low observed pain respectively. In addition, a cue not associated with any pain level, either directly experienced or observed, and that did not appear during the conditioning sessions was introduced as a control stimulus during the test phase when a directly experienced, moderately painful heat stimulus accompanied all five cues. The specific assignment of fractal images to a given condition was fully counterbalanced across participants. All stimuli were presented using the Presentation® software (Version 16.3, www.neurobs.com).

#### Heat pain administration

Noxious heat stimuli were delivered using a PATHWAY system (Medoc Advanced Medical Systems, Israel). All heat stimuli were initiated from a baseline temperature of 32 °C and increased to the target temperature. Each stimulus was presented for 2.5 seconds, including approximately 200 ms to ramp up to the target temperature, maintaining the temperature for 2 seconds and 350 ms to ramp down to the baseline temperature again. Heat stimuli were applied to the right volar forearm; the position of the probe was changed for every session. The mean moderate temperature used during the test phase was 46.5 ± 1.9 **°**C (Mean ± SD). The average difference between the high and low temperatures during the conditioning phase was 3.1 ± 0.9 **°**C.

In order to determine the individually calibrated pain stimuli, an ascending heat pain sequence starting at 35 °C and slowly increasing by 1 °C up to 50 °C or the subject’s maximum tolerance was used. Temperatures that elicited subjective intensity ratings of low = 5, moderate = 10, and high = 15 on a scale^28^ from 0 to 20 were selected for each subject. Once the low, moderate and high heat pain levels had been determined, the subject was tested for rating response consistency. A random sequence of 3 low and 3 high intensity noxious stimuli was administered. When the participant could reliably rate the high stimulus as more intense than the low stimulus, the conditioning phase of the experiment was initiated.

### Experimental procedures

**Figure 1:**
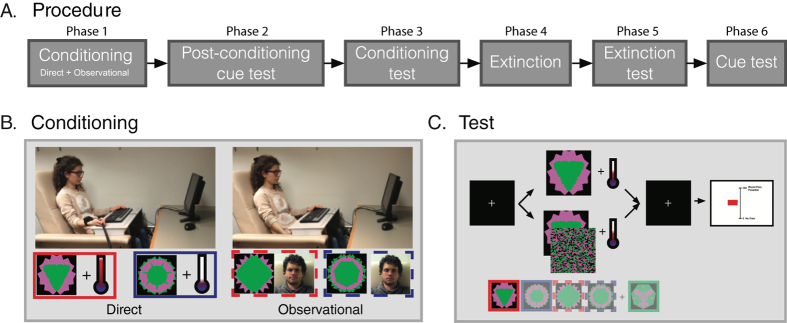
Experimental procedures. (**A**) Succession of experimental phases. (**B)** Conditioning types: direct cues—1 high (red) and l 1ow (blue)—were accompanied by painful stimulation of high or low intensity; observational cues—1 high (red dotted) and 1 low (blue dotted)—were accompanied by observing a model experience high or low level of pain, showing both the physical reaction and subjective pain ratings of the model. Each participant experienced both direct and observational conditioning, learning 4 cues during this phase. (**C)** Stimuli presentation during test. One of the 5 cues (4 cues learned by the subjects during the conditioning phase and 1 new control cue) would appear either supraliminally (200 ms) or subliminally (backward-masked 33 ms + 167 ms), followed by a fixation cross displayed for 4 s. Identical moderate pain (~2.5 s) accompanied all the cues. A rating scale appeared for 7 s, followed by a fixation cross presented between trials for a variable time from 1 to 2 s. Subjects rated each stimulus on a 0–100 scale (VAS).

The experiment consisted of six phases: 1) conditioning (2 sessions of direct and 2 sessions of observational conditioning), 2) post-conditioning test, 3) conditioning test (2 sessions), 4) extinction (1 session), 5) extinction test (1 session), and 6) cue recognition task, Fig. 1A. It took place in 1 day.

During the **conditioning phase (1)**, subjects experienced both direct and observational conditioning. The succession of alternating sessions: ‘direct – observational – direct – observational’ or *vice versa* was counterbalanced across subjects, Fig. 1B.

During the direct conditioning trials, subjects saw two images, each consistently paired with a high or low pain level (100% reinforcement). Subjects were informed that they would see images on the computer screen and that they would experience heat pain stimulation on their arm. Subjects were told that there would be a link between the cue and the pain intensity of the stimulus that they would feel shortly thereafter. There were 10 high and 10 low cue/pain stimulus presentations in a randomized order in each of the 2 sets of trials. After each pain stimulus, the subject rated the pain felt on a 0–100 visual analogue scale (VAS), using a computer mouse and a scale displayed on the screen. The pain ratings for low and high pain were significantly different (p < 0.001); low pain elicited an average rating (Mean ± SD) of 21.2 ± 11.9 and high pain was rated 65.8 ± 12.3 on the 0–100 VAS.

During the observational conditioning sessions, subjects saw two different images that were consistently paired with a high or low pain level, as during the direct conditioning trials. However, subjects did not experience pain directly, but rather watched a video clip in which a model (a research assistant in our lab) experienced a high or low level of pain stimulation to their arm. During the first set of trials, a female model was shown experiencing the pain stimulus; during the second set of trials, a male model was observed. Each trial comprising the video clip began with a side view of the model that also showed the experimental set-up, which included the heat probe placed on the hand, the computer screen, etc. After that, the conditioning cue (high or low) was displayed on the screen; it was followed by a close-up of the face of the model exhibiting a painful grimace following the high pain cue or a neutral expression after the low pain cue. At the end of the video clip, the 0–100 VAS was displayed, which appeared to be scrolled by the model to a lower (20.5 ± 6.3) or higher (73.5 ± 6.5) position on the scale to indicate the model’s pain rating. Subjects were instructed to watch each video clip and learn the association between the cues and pain levels. Similar to the direct conditioning trials, 10 high and 10 low cue/pain pairings were presented in a randomized order in each of the 2 sets of trials.

Following the conditioning phase, during which subjects learned the 4 different cues and their association with a high or low pain stimulus, memory of the cue/pain pairings was tested by presenting all of the cues without heat stimuli or video of the models’ responses and asking subjects to indicate whether each cue was associated with a high or low level of pain. Each of the 4 cues was repeated twice in this **post**–**conditioning phase (2).** Mean accuracy of cue recognition was high (97.5%); only 1 subject did not identify all of the cues correctly.

For the **test phase (3)**, we informed subjects that they would see the 4 cues again along with an additional 5^th^ cue that they had not seen before. Subjects were also told that half of the time the cues would appear fully visible as before (supraliminally) for 200 ms, and that half of the time the cues would appear very briefly (subliminally) followed by a masking image (33 ms cue + 167 ms mask) so that the subject would not be able to recognize the cue (Fig. 1C). All previously conditioned cues were presented concurrently with a moderately painful heat stimulus that was of the same intensity from trial to trial in order to test the effect of the conditioned cue on pain perception. Subjective pain ratings on a 0–100 VAS were collected. There were 2 sets of 30 conditioning test trials; each set began with 2 additional test items, one presented supraliminally and one subliminally, in order to familiarize subjects with the heat stimulus and presentation durations.

Following the conditioning test, subjects underwent an **extinction phase (4).** During this phase, the 4 previously conditioned cues were presented in a random order (5 repetitions per cue) paired with a warm, but not painful, heat stimulus that was the same (34 **°**C) for all subjects. Subjects were presented with the directly and observationally conditioned cues during the extinction phase.

We tested the effects of extinction during the **extinction test phase (5)** by repeating one set of trials as had been presented in the test phase (3) described above.

After the extinction test, subjects underwent the **cue recognition test (6)**, during which they were presented with masked and unmasked cues that were familiar, i.e., previously seen during the experiment, and unfamiliar, i.e., not previously seen, to determine whether they recognized the subliminally presented stimuli. This test was used to confirm that recognition of the subliminal stimuli was at chance level.

In addition to the pain ratings, we also measured skin conductance levels using PowerLab 4SP GSR Amp with bipolar finger electrodes (MLT116F) placed on the distal phalanx of the index and middle fingers of subjects’ non-dominant (left) hand and LabChart v.8.0 software (ADInstruments, Inc., Castle Hill, Australia).

### Statistical analyses

#### Test pain ratings

First, we investigated the effects of direct and observational conditioning with supra- and subliminal cue presentations. We compared subjects’ ratings to the moderately painful heat stimuli that were preceded by the different conditioned cues, both supraliminally and subliminally, using repeated measures ANOVA with factors cue (low vs. high), conditioning type (direct vs. observational) and awareness (conscious vs. non-conscious) and explored significant effects with pairwise comparisons (2-tailed, paired t-tests, FDR-corrected using R ‘p.adjust’ package^29^).

Then we investigated placebo/nocebo responses by examining the neutral cue pain ratings and performing planned comparisons of high vs. neutral and low vs. neutral cues within each type of conditioning (direct and observational) and awareness level (conscious and non-conscious). Four sets of planned comparisons: (1) direct conscious, (2) observational conscious, (3) direct non-conscious and (4) observational non-conscious were performed, using 1-tailed paired t-tests (as the direction of the effect is known *a priori*) adjusted for multiple comparisons using p = 0.05 (robust FDR-adjusted for one-tailed p-values, using R ‘robust-fdr’ package^30^) to determine whether significant placebo/nocebo responses were elicited by each type of conditioning and test presentation.

As half of the subjects were first exposed to direct conditioning and the other half to indirect conditioning, we also compared pain ratings for each type of stimulus between the two groups, using 2-tailed 2-sample t-tests.

#### Skin conductance response analysis

Skin conductance response was calculated as the difference between the peak skin conductance level within the 1–4 s window following cue/pain onset and the average skin conductance level during the 1-second interval before the cue/pain onset. As is commonly done for measures of skin conductance reactivity, a square root transformation was applied to the absolute value of the skin conductance response with replacement of the + or – sign prior to statistical analysis. As done for the primary analyses, we first explored the effect of conditioning type by performing an ANOVA that included awareness (conscious vs. non-conscious), conditioning type (direct vs. observational) and cue (high vs. low) as factors, as well as planned comparisons between high vs. low, high vs. neutral and low vs. neutral cues. We used this early (1–4 s) time window (the so-called first interval response that is considered a reliable measure of skin conductance response^31^,^32^) as we expected it to capture the response to the cue. We also performed the statistical analysis on the skin conductance response over the 1–6 s period in the same way, as the maximum peak latencies for all conditions over the 7 second period (following the cue onset but preceding the pain rating) appeared between 4.5 and 5.3 s, as in other studies using heat pain with comparable temperatures; therefore this wider time window likely reflected the response to pain.

#### Test after extinction

Finally, we compared the conditioning test effect with the effect following the extinction phase. For that we calculated the effect of conditioning as a difference between high and low cue ratings (high minus low) for the conditioning test (phase 3) and for the extinction test (phase 5). We used a repeated measures ANOVA with the factors phase (conditioning test vs. extinction test), conditioning type (direct vs. observational) and awareness (conscious vs. non-conscious).

## Results

### Pain ratings during test

**Figure 2:**
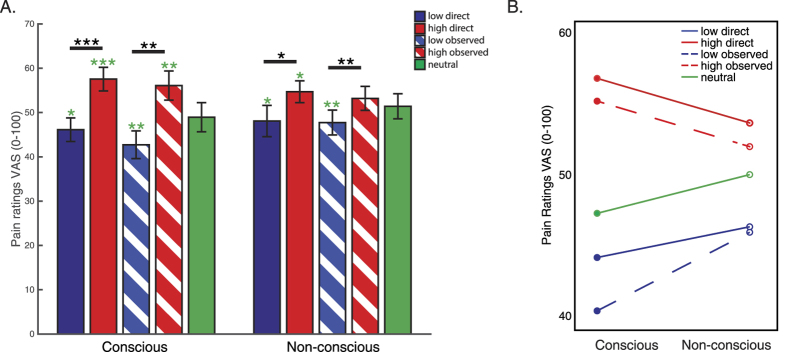
Subjective pain ratings analysis results. (**A**) Subjective pain ratings by awareness (conscious vs. non-conscious) are shown. Black asterisks mark significant differences between ‘high’ and ‘low’ cues in each of the experimental conditions (direct conscious, observational conscious, direct non-conscious, observational non-conscious); 2-tailed paired t-tests, FDR-adjusted for multiple comparisons (***p < 0.001; **p < 0.01; *p < 0.05). Green asterisks mark significant differences between the neutral cue and direct low, direct high, observational low and obervational high cues within each of the awareness levels (conscious vs. non-conscious); 1-tailed paired t-tests, FDR-adjusted for multiple comparisons (***p < 0.001; **p < 0.01; *p < 0.05). (**B)** An interaction plot, cue type by awareness level.

First, we compared subjects’ ratings to identical heat stimuli that were preceded by the respective cues presented subliminally or supraliminally using repeated measures ANOVA with factors: cue (low vs. high), conditioning type (direct vs. observational learning) and awareness (conscious vs. non-conscious). Ratings to the new neutral cue were not included in this analysis. Results revealed a main effect of conditioning type [F_(1,19)_ = 5.136, p = 0.035, η^2^ = 0.213]; pain ratings to directly learned cues were higher (Mean ± SE: 51.6 ± 2.6) than to observationally learned cues (49.9 ± 2.6). Results also showed the main effect of cue [F_(1,19)_ = 23.091, p < 0.001, η^2^ = 0.549] and an interaction of cue and awareness [F_(1,19)_ = 8.855, p < 0.008, η^2^ = 0.318], indicating that the conditioning effects of both direct and observed cues were smaller with subliminal presentation (Fig. 2B). Pairwise comparisons (FDR-corrected) between low and high cues were significant in all four conditions: direct conscious (p_FDR_ < 0.001), observational conscious (p_FDR_ = 0.001), direct non-conscious (p_FDR_ = 0.019), observational non-conscious (p_FDR_ = 0.012); see black asterisks in Fig. 2A. Chance-level recognition of subliminal stimuli (Mean ± SE: 52.92 ± 3.64% correct) was ascertained at the end of the experiment.

In order to determine whether significant placebo/nocebo responses were produced by the respective conditioning and presentation methods, we compared conditioned low and high cues to the neutral conscious and non-conscious cues. Significant placebo and nocebo responses were observed with both directly and observationally learned supraliminally presented cues (low direct vs. neutral, p_FDR_ = 0.042; high direct vs. neutral, p_FDR_ = 0.001; low observational vs. neutral, p_FDR_ = 0.007; high observational vs. neutral, p_FDR_ = 0.015). With subliminally presented cues, both placebo and nocebo effects were observed with directly learned cues (low direct vs. neutral, p_FDR_ = 0.027; high direct vs. neutral, p_FDR_ = 0.033). However, with the observationally learned cues, only the placebo effect was significant (low observational vs. neutral, p_FDR_ = 0.003; high observational vs. neutral, p_FDR_ = 0.128); see green asterisks in Fig. 2A.

No differences between the pain ratings of the subjects who experienced direct conditioning first and those who experienced observational conditioning first were observed for any of the stimuli.

### Skin conductance responses during test

**Figure 3:**
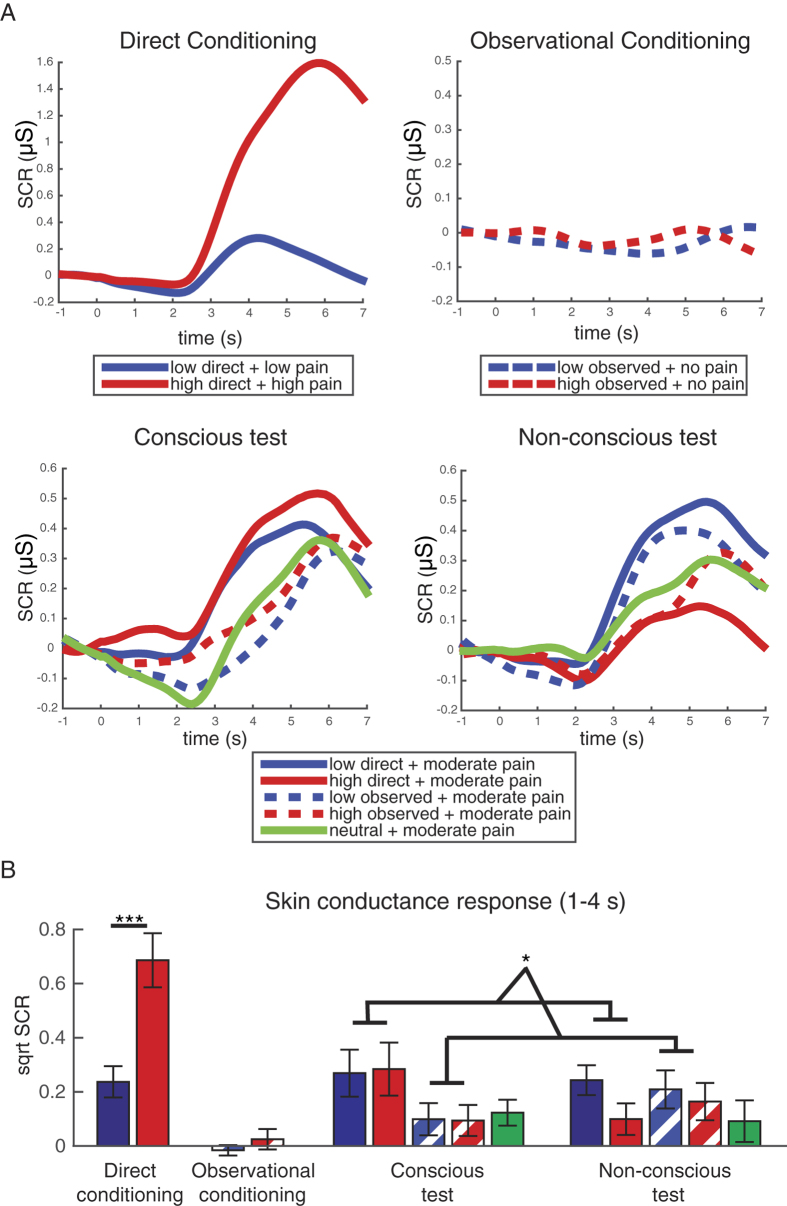
Skin conductance response (SCR) analysis results. (**A**) Baseline-corrected skin conductance time-locked to the onset of the cue (−1 to 7 seconds). Top left: direct conditioning showing the response to the cue accompanied by low and high pain. Top right: observational conditioning showing the response to the cue accompanied by observed pain. Botton left: test results showing the response to the supraliminally presented cue accompanied by identical moderate pain. Bottom right: test results showing the response to the subliminally presented cue accompanied by identical moderate pain. (**B)** Mean SCR calculated as the square root of the maximum over 1–4 s after the stimulus onset minus the average value in the −1–0 s period for direct conditioning, observational conditioning, conscious test, non-conscious test; error bars represent standard errors (SEs). The results for the different experimental phases are plotted using the same scale to facilitate direct comparison.

We also examined skin conductance responses during conditioning and test phases (Fig. 3). We focused on the first response interval 1–4 s after the cue onset. A difference between low and high cues was observed during direct conditioning (p < 0.001) but not observational conditioning (p = 0.27). During test, results of repeated measures ANOVA, as described above, revealed a significant main effect of conditioning type [F_(1,19)_ = 5.576, p = 0.029, η^2^ = 0.227]. There were larger skin conductance responses to directly (Mean ± SE: 0.22 ± 0.06), compared to observationally, learned cues. There were no significant differences between low and high cues or differences from the neutral cues during the test phase.

The repeated measures ANOVA for the SCR in the wider 1-6 s time window showed the main effect of awareness [F(1,19) = 10.377, p = 0.004, η2 = 0.353]. Larger skin conductance responses were observed with supraliminal (Mean ± SE, 0.40 ± 0.08), compared to the subliminal (0.31 ± 0.07), presentations. No differences between cues or conditioning types were observed in this wider time window.

### Test after extinction

**Figure 4:**
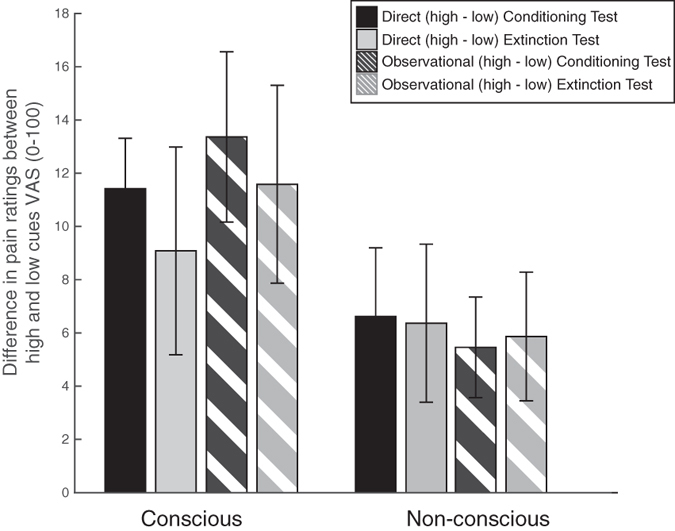
Extinction analysis results. The difference in pain ratings between high and low cues for each of the experimental conditions (direct conscious, observational conscious, direct non-conscious, observational non-conscious) by phase—Conditioning test (black) vs. Extinction test (gray).

In order to determine whether extinction (20 trials) would affect direct/observational or conscious/non-conscious results differentially, we compared the initial test (phase 3) with the test (phase 5) following extinction (Fig. 1A), during which all previously learned cues were presented with a non-painful, warm stimulus. Differences between reported pain for high and low cues were calculated (high minus low) and examined in a repeated measures ANOVA with the factors phase (conditioning test vs. extinction test), conditioning type (direct vs. observational) and awareness (conscious vs. non-conscious). Results revealed a main effect of awareness [F_(1,19)_ = 34.641, p = 0.033, η^2^ = 0.217]. This suggests that while the differences between high and low cue responses were smaller for subliminal compared to supraliminal presentation, they were not different between conditioning types or phases (Fig. 4).

## Discussion

We compared the effect of supraliminal and subliminal presentation of directly and observationally conditioned cues on pain perception. Although directly conditioned cues generally produced greater pain sensitivity as evidenced by larger skin conductance responses and higher pain ratings, the magnitude of the conditioning effect during test phases was similar for directly and observationally conditioned cues. Importantly, significant differences between low and high cues presented subliminally were observed for both directly and observationally conditioned cues. Our subliminal effects appeared to be modest but comparable to a recent study employing a similar direct conditioning paradigm^38^.

### Direct and indirect exposure to cues produce different experiences in pain perception but the same pain modulation

**Figure 5:**
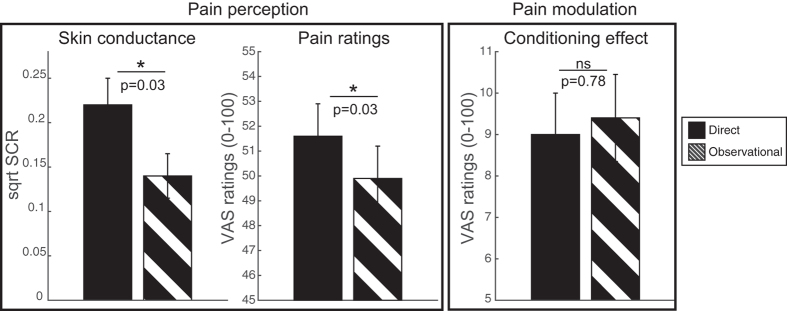
Main effect of conditioning type (direct vs. observational) results summary. Direct vs. observational conditioning (across conscious and non-conscious cues) for a) skin conductance response (1–4 s time window), b) pain ratings, c) conditioning effect (high minus low cue rating) during test. Significant main effects of conditioning type within 3-way ANOVA (conditioning type by awareness by cue) are discussed in the main text of the manuscript. Here p-values of 2-tailed t-tests are provided. The results indicate lower arousal and pain intensity in response to observational cues compared to direct cues, but equal conditioning effect obtained through direct and observational learning.

Studies investigating autonomic responses to observational conditioning have found direct, compared to observational, learning to produce larger skin conductance responses^39^, as well as greater heart rate deceleration to conditioned placebo stimuli^22^. In contrast, a study that used social observation to establish placebo and nocebo learning and measured subjective pain ratings found no difference in the amplitude of the placebo effect between direct and observational conditioning^23^. Our findings help to reconcile the discrepancy between autonomic reactivity and pain rating effects. Direct and observational conditioning both produced significant differences between the high and low cues (pain modulation). However, observational conditioning was associated with lower pain ratings and smaller skin conductance responses, as suggested by a significant main effect of conditioning type in pain ratings and early skin conductance response. These results are illustrated in Fig. 5. This dissociation between pain perception (lower autonomic arousal and perceived pain intensity) and pain modulation (equal placebo and nocebo effects) suggests that observational conditioning might be a good alternative way of inducing placebo/nocebo, as pain is not directly experienced. While we observed statistically significant differences between the conditioning types in this first within-subject study comparing direct and observational effects, the differences were subtle, much smaller than the reported placebo/nocebo effects. Observed differences in pain perception therefore warrant further investigation and replication.

### First-hand experience with cues and awareness of cues is not necessary to produce placebo and nocebo effects

Previous studies investigating non-conscious activation of directly and observationally conditioned cues used emotional stimuli (painful images or fearful faces)^4^,^40^. Depending on the measure, conscious awareness was found to be either not necessary (to produce a skin conductance response to emotional cues^40^), or necessary (to produce subjective hyperalgesia, i.e., modulate pain ratings^4^). The results of a recent study^38^ examining conscious and non-conscious cue learning and activation through direct exposure to pain suggested that conscious awareness is not necessary to induce placebo and nocebo. The current study is the first to demonstrate that learning through social observation can also produce a change in subjective pain ratings even with subliminally presented cues. This suggests that neither direct learning nor conscious awareness is necessary to elicit a significant change in pain experience. The effects of subliminal presentation in our study were smaller than those achieved with supraliminal cues, which could be related to the use of abstract visual cues rather than faces and pain-related images used in previous studies.

### The effects of observational learning, both conscious and non-conscious, withstand extinction

The direct and observational conditioning effects persisted following extinction trials, suggesting that these conditioning effects are equally durable (Fig. 4). The subliminal effects were smaller, but also did not extinguish. The lack of extinction has been previously reported in placebo^19^, as well as classical and observational fear conditioning^40^ studies. Given the number of extinction trials, this experimental phase only served to demonstrate the robustness of conditioned placebo and nocebo effects rather than eliminate them. This result provides further support for the potential use of social observation methods for pain modulation.

### Study Limitations

Although the within-subject design allowed us to examine the effects of direct and observational learning within the same individual, it also meant that all subjects had experience with the direct conditioning paradigm, as direct and observational sessions alternated. The direct conditioning experience could have influenced observational learning, perhaps by making the experience of the model in the video easier to imagine. However, previous work has found that the effect of observational conditioning is attenuated following exposure to direct conditioning, rather than augmented^41^. We observed equally strong conditioning for direct and observational conditioning, and our results were comparable to previous between-group studies^22^,^23^.

## Additional Information

**How to cite this article**: Egorova, N. *et al.* Not seeing or feeling is still believing: conscious and non-conscious pain modulation after direct and observational learning. *Sci. Rep.* **5**, 16809; doi: 10.1038/srep16809 (2015).
